# Splenic Artery Embolism in Liver Transplant Patients: A Single-Center Experience

**DOI:** 10.7759/cureus.38599

**Published:** 2023-05-05

**Authors:** Mark S Obri, Wasih Kamran, Mohamed Ramzi Almajed, Daniel Eid, Deepak Venkat

**Affiliations:** 1 Internal Medicine, Henry Ford Health System, Detroit, USA; 2 Radiology, Henry Ford Health System, Detroit, USA; 3 College of Medicine, Northeast Ohio Medical University, Detroit, USA; 4 Division of Gastroenterology and Hepatology, Henry Ford Health System, Detroit, USA

**Keywords:** spleen, complications, interventional radiology guided embolization, transplant, hepatology

## Abstract

Background: Hypersplenism, portal hypertension, and ascites have been seen after liver transplants. Patients are usually treated medically with refractory patients potentially undergoing splenectomy. Splenic artery embolism (SAE) is an alternative that can be performed to limit the surgical intervention that may have the benefit of improving portal hypertension. Few studies have studied the effect on main portal vein (MPV) velocities and hepatic artery resistive indices (HARIs) which may be beneficial as markers of portal hypertension.

Purpose: This study aims to evaluate the efficacy and safety of interventional radiology (IR)-guided SAE for the management of portal hypertension in patients who have had liver transplants.

Methods: A retrospective analysis was conducted on liver transplant patients who had undergone IR-guided SAE post-transplant at a single tertiary transplant center from 2012 to 2022. The primary outcome of intervention efficacy was quantified by peak HARIs and MPV velocities. Ultrasound with Doppler obtained before and after the intervention was reviewed for these parameters. Secondary outcomes included adverse events at the time of the procedure and within one year of the procedure, the need for splenectomy, and spleen size.

Results: Twenty-eight patients met the criteria for inclusion. The mean age of patients was 52.5 years (21-71 years) and the time after transplant was 149.5 days (2-1588 days). About 96.4% of SAEs were technically successful (n=27). Twenty-one patients had MPV velocities available, and 24 had peak HARIs available. In these patients, HARIs decreased by an average of 0.063 (95% CI 0.014-0.112) after SAE. MPV velocity decreased by an average of 47.2 cm/s (95% CI 27.3-67.1) after SAE. About 10.4% of patients (n=3) developed a procedure-related complication, all of which were femoral access site aneurysms. No (0) patients suffered from bleeding, infections, or abscesses after the procedure. About 10.7% of patients (n=3) required splenectomy after SAE: one splenectomy was due to technical failure and two were due to refractory symptoms.

Conclusion: We performed one of the first analyses on MPV and RI and showed that our patients saw an improvement post-embolization with a theoretical improvement in portal hypertension. The complication rate and risk of infection seem to be acceptable risks, making SAE a feasible option for management.

## Introduction

Splenic artery embolization (SAE) was first described by Maddison in 1973 when he performed a complete splenic artery embolization using an autologous clot on a presumed cirrhotic patient suffering from recurrent esophageal variceal bleeding [1]. Since then, SAE has been performed for a myriad of conditions including trauma, hypersplenism, portal hypertension, and splenic artery aneurysm [2]. More recently, partial SAE has been performed in patients with cirrhosis to aid in the treatment of multiple sequelae of cirrhosis including thrombocytopenia, hypersplenism, and portal hypertension [3]. The breadth of indications has allowed ample data to be published regarding the adverse outcomes of SAE. These include splenic rupture, abscess, splenic vein thrombosis, pleural effusion, and pancreatitis [3-5].

SAE has additionally been described in the treatment of portal hyperperfusion in orthotopic liver transplant (OLT) patients. Portal hyperperfusion is a phenomenon wherein increased portal pressures post-transplant result in reciprocal decreases in hepatic arterial flow, manifesting as high resistive indices (RIs) on liver ultrasounds. The clinical manifestations of hyperperfusion include refractory ascites, hypersplenism, and graft dysfunction [6]. Additionally, patients who have received OLT frequently demonstrate early increases of hepatic artery RIs (HARIs), which in turn rise in the presence of surrogate markers indicating portal hypertension [7]. This is suggestive of a possible correlation between HARIs and portal hypertension.

SAE has been proven to be efficacious in decreasing HARIs and splenic volumes in OLT patients when compared to matched controls [8]. While these parameters are excellent secondary markers of portal hyperperfusion, there is not adequate data on changes in main portal vein (MPV) peak velocity, a more direct measure of portal pressure. Here, we describe the clinical and hemodynamic outcomes of patients who underwent OLT and subsequent SAEs between 2012 and 2022 at a single liver transplant center.

## Materials and methods

Methods

This is a single-center retrospective study of liver transplant patients who underwent interventional radiology-guided splenic artery embolism (SAE) after transplant at a tertiary transplant center from 2012 to 2022. Patients who underwent SAE prior to liver transplantation and those who underwent SAE in the context of other disease conditions (idiopathic thrombocytopenic purpura and splenic trauma) were excluded. Patients that had missing information were excluded.

Indications for SAE

Among liver transplant patients, SAE has shown promising results in mitigating complications related to the hemodynamic and hematologic changes that subsequently occur. Based on published literature, indications for SAE in our patient population was stratified into thrombocytopenia, splenic steal syndrome, portal hypertension, and other; the other classification was used to group less-common justifications for SAE which included splenic artery aneurysms, splenic artery pseudoaneurysms, and graft dysfunction. Thrombocytopenia was defined as a platelet count of less than 150 K/uL. Splenic steal syndrome was defined as a graft dysfunction in the setting of sluggish hepatic artery flow without significant artery obstruction; this is caused by increased blood flow to a dominant splenic artery that siphons flow away from the hepatic artery. Portal hypertension was defined as an elevated hepatic venous pressure gradient with clinical manifestations of increased portal pressure.

Study outcomes

The primary outcome was the intervention efficacy as quantified by peak HARIs and MPV velocities; a decrease in these two objective measures was consistent with treatment efficacy. Ultrasound of the liver with color and spectral Doppler was performed before and after SAE to quantify these parameters. Secondary outcomes included procedure technical success, spleen size, platelet count, white blood cell count, and subsequent need for splenectomy. Patients with at least six months of follow-up also were measured for the rate of liver biopsy-proven rejection, bacterial infection, and *Cytomegalovirus* (CMV) viremia. Patient charts were reviewed up to the time of the last outpatient follow-up with hepatology.

Statistical analysis

Data was extracted from a review of the electronic medical record of all SAE procedures performed from 2012 to 2022 followed by stratification to limit the search to patients who underwent liver transplantation. Technical success, means, and percent change in study outcomes were calculated.

## Results

Population

The relevant demographic data was included in Table 1.

**Table 1:** Demographic data of population SAE: splenic artery embolism

Demographic data	
Mean age	52.5 (21-71) years
Sex: male	18 (64.3%)
White race	28 (100%)
Mean time to SAE	149.5 days (2-1588 days)

Technical success

Of the 28 patients, 27 demonstrated technical success (96.4%). One procedure was technically unsuccessful due to the reported difficult and tortuous anatomy. About 10.7 % of patients (n=3) developed a procedure-related complication, all of which were femoral access site aneurysms. No (0) patients suffered from bleeding, infections, or abscesses after the procedure. About 10.7% of patients (n=3) required splenectomy after SAE: one splenectomy was due to technical failure and two were due to refractory symptoms.

MPV velocities

Twenty-one patients had MPV velocities available. MPV velocity decreased by an average of 47.2 cm/s (95% CI 27.3-67.1) after SAE.

HARIs

Twenty-four patients had HARIs available. In these patients, HARIs decreased by an average of 0.063 (95% CI 0.014-0.112) after SAE.

Rates of rejection

Twenty-two of the 28 patients had information available at six months of follow-up. Of these patients, 13.6% (n=3) had rejection. All rates of rejection were confirmed on liver biopsy.

Infections after embolism

Twenty-two patients had six-month follow-up data available. Of those patients, three patients had bacterial infections within a six-month period. These infections included cholangitis, *Clostridium difficile*, and a non-splenic abscess. Two other patients had a viral infection within six months and presented with CMV viremia.

Spleen size

Twenty-eight patients had available data on spleen size before and after SAE. The average change in spleen size was a decrease of 1.07 cm (0.78-1.36).

## Discussion

The findings of this study suggest that SAE is a safe and efficacious procedure both during and in a six-month interval after the procedure. Our study is one of the first to demonstrate a decrease in main portal vein peak velocity post-SAE in OLT patients. While previous studies have demonstrated a decrease in HARIs, very few have shown a direct effect on portal venous flow after SAE. One study had been conducted in which a decrease in MPV peak velocity was shown in four patients. However, this was limited by the small number of patients [5]. Our study showed an average decrease of 47.2 cm/s following SAE. This data can be useful for allowing future studies to compare decreases in MPV to clinical outcomes in transplant patients. With high portal venous being a primary cause of portal hyperperfusion, it can be used as a measure to supplement HARIs. Potentially reinforcing this data with a more upstream measurement further solidifies the utility of SAE in decreasing portal hyperperfusion.

Our study also demonstrates that SAE can decrease RIs. This data is in line with other studies that have revealed similar findings in improved RIs and hepatic arterial blood flow [7-10]. As mentioned, RIs frequently increase following an OLT, notably in the setting of portal hypertension [7]. This data can be used to encourage physicians to potentially refer SAE patients with refractory portal hypertension. In addition, SAE would be an alternative to splenectomy which is more invasive and potentially can have more complications. A few of our patients failed SAE requiring further splenectomy. Therefore, it appears to be a reasonable alternative to an open splenectomy.

While showing the benefit of SAE, it is important to note that there is a theoretical risk of infection and rejection after embolizing the spleen. We observed a rejection rate of 13.6% in our study population which is comparable to baseline liver transplants in the setting of patients who have not received SAE [9]. We also noted that our patients had three recorded episodes of bacterial infections and two cases of CMV viremia. The clinical significance of this is unclear due to the small number of patients but appears acceptable given the immunocompromised status of liver transplant patients. This study can potentially be extrapolated to other portal hypertension patients with concurrent measurements of rates of paracentesis, thoracentesis, and other markers to measure improvement in portal hypertension.

Our study is limited by the small number of procedures due to the relative rarity of an SAE. Because of its retrospective nature, it is nonrandomized, and the indications for SAE across the patients are not uniform. The study is also limited by our significant reliance on hepatology/transplant notes rather than objective documentation due to the nature of this study (i.e., rejection rates and complications). When available, we attempted to look at pathology reports, but oftentimes, procedures were done in a variety of different hospitals with minimal record access.

## Conclusions

Splenic artery embolization is a safe and effective technique for lowering patients’ HARIs as well as their main portal peak velocity. This is promising for patients who require (SAE) for indications such as portal hypertension, refractory ascites, and splenomegaly and can potentially be an alternative to open splenectomy. Larger, multi-center studies will be required to assess further long-term data and effectiveness as well as measure the correlation between MPV and clinical improvement.
